# Effect of intraoperative remimazolam on postoperative sleep quality in elderly patients after total joint arthroplasty: a randomized control trial

**DOI:** 10.1007/s00540-023-03193-5

**Published:** 2023-04-13

**Authors:** Chun-Mei Deng, Zhao-Ting Meng, Jing Yang, Cai-Juan Zhang, Min Lu, Yue-Xin Wang, Dong-Liang Mu

**Affiliations:** 1 Department of Anesthesiology, Peking University First Hospital, No. 8 Xishiku Street, Beijing, 100034 China; 2 Department of Anesthesiology, Cangzhou People’s Hospital, Cangzhou, Hebei China; 3 Department of Anesthesiology, Tangshan Gongren Hospital, Tangshan, Hebei China

**Keywords:** Bispectral index, Remimazolam, Sedation, Sleep, Total joint arthroplasty

## Abstract

**Purpose:**

To investigate the effect of intraoperative remimazolam sedation on postoperative sleep quality in elderly patients after total joint arthroplasty.

**Methods:**

Between May 15, 2021 and March 26, 2022, 108 elderly patients (age ≥ 65 years) who received total joint arthroplasty under neuraxial anesthesia were randomized into remimazolam group (a loading dose of 0.025–0.1 mg/kg and followed by an infusion rate of 0.1–1.0 mg/kg/h till end of surgery) or routine group (sedation was given on patient’s requirement by dexmedetomidine 0.2–0.7 μg/kg/h). Primary outcome was the subjective sleep quality at surgery night which was evaluated by Richards Campbell Sleep Questionnaire (RCSQ). Secondary outcomes included RCSQ scores at postoperative first and second nights and numeric rating scale pain intensity within first 3 days after surgery.

**Results:**

RCSQ score at surgery night was 59 (28, 75) in remimazolam group which was comparable with 53 (28, 67) in routine group (median difference 6, 95% CI − 6 to 16, *P* = 0.315). After adjustment of confounders, preoperative high Pittsburg sleep quality index was associated worse RCSQ score (*P* = 0.032), but not remimazolam (*P* = 0.754). RCSQ score at postoperative first night [69 (56, 85) vs. 70 (54, 80), *P* = 0.472] and second night [80 (68, 87) vs. 76 (64, 84), *P* = 0.066] were equivalent between two groups. Safety outcomes were comparable between the two groups.

**Conclusions:**

Intraoperative remimazolam did not significantly improve postoperative sleep quality in elderly patients undergoing total joint arthroplasty. But it is proved to be effective and safe for moderate sedation in these patients.

**Clinical trial number and registry URL:**

ChiCTR2000041286 (www.chictr.org.cn).

**Supplementary Information:**

The online version contains supplementary material available at 10.1007/s00540-023-03193-5.

## Introduction

Sleep disturbance frequently occurs in elderly patients after surgery due to perioperative anxiety, stress and postoperative pain [1]. In patients undergoing total joint arthroplasty, the reported incidence of sleep disturbance reaches up to 60% [2, 3]. Many studies have demonstrated that postoperative sleep disturbances are associated with worse clinical outcomes including increased risk of postoperative complications, prolonged duration of in-hospital stay, and poor quality of recovery [4].

Neuraxial anesthesia is commonly used for patients undergoing total joint arthroplasty [5]. These patients usually suffer from moderate to severe anxiety and pain which are considered as major risk factors of perioperative sleep disturbance [2, 3, 6]. Sedation during neuraxial anesthesia is an effective approach to alleviate intraoperative anxiety. Intraoperative sedatives, such as dexmedetomidine [7] and midazolam [8], are also reported to improve postoperative sleep quality. Furthermore, one randomized trial showed that intraoperative midazolam with bispectral index (BIS) around 77 provides better sleep quality than dexmedetomidine in patients after elective transurethral prostatic resection [8]. Yet, deficits of midazolam include a relative long-acting time for two to three hours and the potential risk of delirium [9]. Remimazolam is an ultra-short-acting benzodiazepine which takes significant advantage in shorter elimination half-life of several minutes and better recovery of cognitive function from procedure sedation in comparison with midazolam [10]. Up to now, there is little data to elucidate the relationship between intraoperative infusion of remimazolam and postoperative sleep quality.

Present study was designed to investigate the effect of intraoperative remimazolam for sedation on sleep quality at surgery night in elderly patients after total joint arthroplasty. We hypothesized that intraoperative sedation with remimazolam could improve sleep quality at surgery night compared with routine care group.

## Methods

### Participants

This randomized trial was approved by Biomedical Research Ethics Committee of Peking University First Hospital (No. 2020-350, Chairperson Prof. Yanyan Yu on December 15, 2020) and registered at Chinese Clinical Trial Registry (www.chictr.org.cn; No. ChiCTR2000041286; December 23, 2020). The study was conducted in Peking University First Hospital and Cangzhou People's Hospital. Written informed consents were obtained from all participants.

Elderly patients, aged 65 years old or above, who were scheduled for elective total joint arthroplasty under neuraxial anesthesia were enrolled. Patients were excluded if they met any of the following criteria: (1) allergy to remimazolam or dexmedetomidine; (2) sleep disorder requiring medical interventions (i.e., hypnotics) within recent 1 month; (3) Severe arrythmia including sick sinus syndrome, severe bradycardia (heart rate < 50 beats per minute), or atrioventricular block of second degree or above without pacemaker; (4) severe renal dysfunction (requiring renal replacement therapy); (5) Child–Pugh class C; and (6) American Society of Anesthesiologists physical status (ASA-PS) ≥ IV.

### Randomization and group allocation

Patients were randomized into either remimazolam group or routine group at 1:1 ratio with a block size of 4 and stratified by study centers by an independent biostatistician (SAS 9.2. SAS Institute, Cary, NC). Random numbers were sealed in sequentially numbered opaque envelopes and stored at study centres until the end of the study. A researcher was designated to allocate random number 30 min before anesthesia and prepared trial drugs per protocol. This researcher was not involved in administration of intervention, follow-up, and data collection.

### Anaesthesia and interventions

No premedication was given prior to surgery. All patients received standard monitoring including heart rate and rhythm, non-invasive blood pressure, arterial pulse saturation (SpO_2_), and Bispectral index (BIS). L2-3 or L3-4 was selected for neuraxial anaesthesia and 0.5% hyperbaric bupivacaine with a volume of 2–3 ml was given for spinal anaesthesia. Epidural catheterization was conducted if expected duration of surgery would be longer than 2 h. Routinely, methylprednisolone 40 mg was given before anaesthesia and tropisetron 5 mg was given at end of surgery.

After confirmation of absolute anaesthesia level above T10, trial drugs were administrated by attending anaesthesiologists. In remimazolam group, a loading dose of remimazolam 0.025–0.1 mg/kg was initially given until the loss of consciousness, and then sedation depth was maintained at BIS 70–80 by continuous infusion at a rate of 0.1–1.0 mg/kg/h till end of surgery. Attending anaesthesiologist could adjust the infusion rate to maintain targeted sedation depth. Additional dosage of remimazolam 0.025–0.1 mg/kg could be given in necessary if the target BIS was not obtained at maximum infusion rate. In routine group, sedation was given at the discrete of patient’s demand with dexmedetomidine (i.e., 0.2–0.7 ug/kg/h) based on routine care. Since the BIS value was not routinely used for monitoring sedation in clinical setting, the readings of BIS value were blinded to attending anaesthesiologists in these patients.

To decrease potential bias, the anaesthesiologists did not take part in enrolment, randomization, postoperative follow-up and data collection. Any exchange of information above was not allowed during the study period. Blind method was also conducted to researchers, patients, and other related healthcare providers. Emergency unmasking of randomization could be taken in terms of severe adverse events which might result in prolonged in-hospital stay, increased medical expense, disability, and death.

### Postoperative analgesia and follow-up

Multimodal analgesia was provided to keep the numeric rating pain score at rest (NRS, a 11-score scale, 0 indicating no pain and 10 for worst pain) less than 3. First, patient controlled intravenous analgesia (PCIA) pump was provided. It was programmed to deliver background infusion of sufentanil at a rate of 1 μg/h and a bolus of 2 μg sufentanil on demand with 8 min lockout time. Second, non-steroid anti-inflammatory drugs (NSAIDs, flurbiprofen 50 mg, parecoxib 40 mg or ketorolac 30 mg) was initially given at end of surgery and then at 12 h interval until postoperative 72 h. Third, ultrasound guided femoral nerve block for knee surgery or fascia iliaca compartment block for hip surgery was conducted with a single injection of 0.3% ropivacaine 20 ml.

Patients were visited twice daily (07:00–09:00 and 19:00–21:00, respectively) during postoperative first 3 days and then once daily during in-hospital stay. After discharge, patients were interviewed by telephone at postoperative 30 days.

### Data collection and outcome assessment

Baseline variables such as demographic data, comorbidities, and major laboratory tests were collected. Sleep quality within recent 1 month was assessed by Pittsburgh Sleep Quality Index (PSQI, ranges from 0 to 21 with higher scores indicating worse sleep quality) [11]. Chronic pain was assessed by the Brief Pain Inventory (BPI, the mean score ranges from 0 to 10 with higher score indicating heavier intensity or worse pain-related function) [12]. Activity of daily living was assessed with the Barthel Index (ranges from 0 to 100 with higher score indicating better daily activity). Anxiety was assessed by Self-Rating Anxiety Scale (SAS, ranges from 20 to 80 with higher score for heavier anxiety) [13].

#### Primary outcome

Primary outcome was the subjective sleep quality at surgery night, assessed by the Chinese version Richards Campbell Sleep Questionnaire (RCSQ) at 07:00–09:00 on postoperative first morning [14, 15].

The RCSQ involves five domains including sleep depth, sleep latency, awakenings, returning to sleep, and overall sleep quality. Each domain is assessed by using a 100-mm visual analog scale with higher scores indicating better sleep. Overall RCSQ sleep score is defined as the mean value of above five domains and classified into four groups: very poor sleep with scores of 1–25, poor sleep with scores of 26–50, good sleep with scores of 51–75, and very good sleep with scores of 76–100. Although originally designed for critically ill patients in intensive care unit, the RCSQ has also been used among general surgical patients [16]. To improve the consistency of sleep assessment, the investigators who performed RCSQ was trained before study beginning and two times during study.

#### Secondary outcomes

RCSQ scores at postoperative first and second nights were assessed in line with primary outcome. NRS pain intensity and the incidence of postoperative nausea and vomiting (PONV) within postoperative first 3 days were recorded. Duration of postoperative in-hospital stay was defined as the interval between surgery day and discharge of hospital. Major complications requiring medical interventions (i.e., Clavien–Dindo classification 2 and above [17]) within postoperative 30 days were also collected.

#### Safety outcomes

Safety outcomes were monitored from administration of study drugs until 6 h after surgery including hypotension, hypertension, bradycardia, and desaturation. We also recorded the occurrence of intraoperative and early postoperative nausea and vomiting. Interventions of above adverse events were conducted according to routine practice.

### Statistical analysis

#### Sample size calculation

In a pilot observation of 20 elderly patients, mean RCSQ score at surgery night was 50 with standard deviation (SD) of 15. We assumed that intraoperative remimazolam would increase the RCSQ score to 60 at surgery night. With statistical significance at 0.05 and power at 90%, the sample size required to detect the difference was 49 patients in each group (PASS 15.0, StataCorp. LP, College Station, TX, USA). Taking a dropout rate of 10%, we planned to enrol 54 patients in each group.

#### Data analysis

Continuous data with normal distribution were expressed as the mean (SD) and analyzed using independent samples t test, whereas data without normal distribution were expressed as median (interquartile range, IQR) and compared by Mann–Whitney *U*-test. Categorical data were presented as number (percentage) and compared by Chi-square test or Fisher’s exact test.

Primary outcome was analyzed in intention-to-treat and per protocol populations respectively. RCSQ score was presented as median (IQR) and compared by Mann–Whitney *U*-test. Median difference and 95% confidence interval (CI) were calculated with Hodges-Lehmann estimator. Multivariable generalized linear model was employed to investigate the association between interventions and RCSQ with adjustment of confounders including unbalanced variables between two groups and clinically important factors. A post-hoc analysis was administrated to compare the RCSQ between patients who received remimazolam and those treated with dexmedetomidine. Categorical outcomes were compared by Chi-square and estimated effect was presented as relative risk and 95% CI. Length of postoperative in-hospital stay was presented as median (IQR) and analyzed using the Kaplan–Meier survival analysis with log-rank test and hazard ratio (HR) calculated by Cox regression analysis.

BIS value was recorded at 1-min interval from initial administration of trial drug to end of surgery. Time-weighted average (TWA) BIS was calculated as the summation of BIS values multiplied by the referred time, and divided by the specified recording duration.

All tests were two tailed and *P* < 0.05 was considered as statistically significant. Statistical analyses were performed with the SPSS 25.0 software (SPSS, Inc., Chicago, IL) and Python 3.7.0 software (Python Software Foundation, Beaverton, OR, USA).

## Results

### Participants

**Fig. 1:**
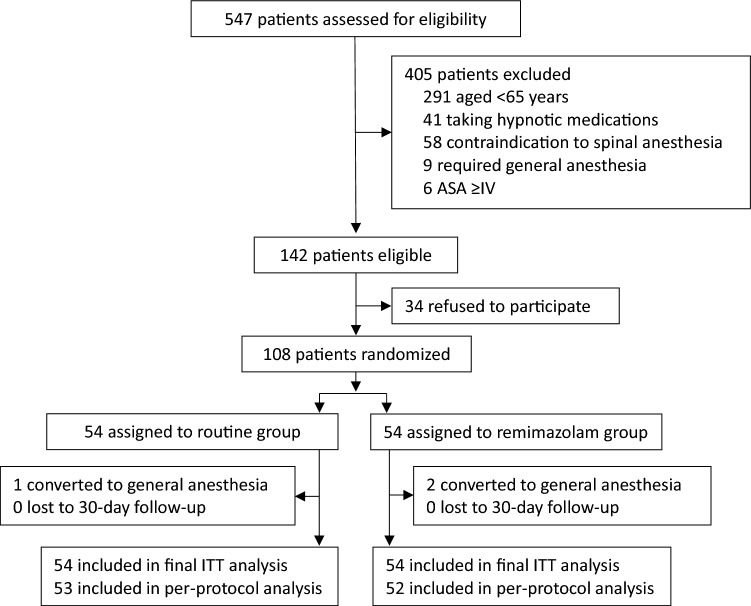
Flowchart of the trial. ITT, intention-to-treat

From May 15, 2021 to February 24, 2022, 547 patients were screened and 108 eligible patients were randomized (Fig. 1). The last follow-up was performed on March 26, 2022. Two patients in remimazolam group and one in routine group received general anaesthesia after randomization because of failure of spinal anaesthesia. In remimazolam group, 52 patients received remimazolam with a median dosage of 25.0 (20.0, 31.3) mg. In routine group, 83.3% (45/54) received dexmedetomidine with a median dosage of 35.0 (25.5, 50.0) μg. No patient was lost or died during 30-day follow-up.

Baseline variables including demographic data and comorbidities were listed in Table 1. Patients in remimazolam group had equivalent PSQI [6.5 (3.3) vs. 7.7 (3.5), *P* = 0.062], SAS [27 (25, 31) vs. 30 (26, 33), *P* = 0.061], and BPI severity scores [2.8 (1.2) vs. 3.1 (1.3), *P* = 0.252] in comparison with routine group. The incidence of hypertension was lower in remimazolam group than routine group (55.6% vs. 77.8%, *P* = 0.014). Perioperative variables were comparable between two groups, except higher percentage of total knee arthroplasty (90.7% vs. 75.9%, *P* = 0.039) and slightly lower TWA BIS [77 (7) vs. 85 (9), *P* < 0.001] in remimazolam group (Table 2). No additional narcotics or benzodiazepines were administered to any patient during surgery.

**Table 1:** Baseline variables

	Remimazolam group (*n* = 54)	Routine group (*n* = 54)	*P*
Age, year	70.8 ± 4.4	71.8 ± 5.5	0.292
Female, *n*	39 (72.2%)	40 (74.1%)	0.828
Body mass index, kg/m^2^	26.3 ± 3.6	26.2 ± 2.5	0.869
Duration of education, year	9 (3, 12)	8 (3, 12)	0.758
Preoperative comorbidities, *n*			
Hypertension	30 (55.6%)	42 (77.8%)	0.014
Coronary heart disease	13 (24.1%)	9 (16.7%)	0.339
Arrhythmia^a^	2 (3.7%)	3 (5.6%)	> 0.99
Stroke	2 (3.7%)	6 (11.1%)	0.270
Diabetes mellitus	10 (18.5%)	18 (33.3%)	0.079
Chronic obstructive pulmonary disease	1 (1.9%)	2 (3.7%)	> 0.99
Alcoholism^b^	2 (3.7%)	3 (5.6%)	> 0.99
ASA-PS classification, *n*			0.430
II	31 (57.4%)	35 (64.8%)	
III	23 (42.6%)	19 (35.2%)	
Pittsburgh sleep quality index, score^c^	6.5 ± 3.3	7.7 ± 3.5	0.062
Self-rating anxiety scale, score^d^	27 (25, 31)	30 (26, 33)	0.061
Brief Pain Inventory, score^e^			
Severity score	2.8 ± 1.2	3.1 ± 1.3	0.252
Interference score	3.6 ± 1.5	3.8 ± 1.4	0.553
Barthel Index, score^f^	95 (90, 100)	95 (85, 100)	0.053

Data are presented as mean ± SD, median (interquartile range), or number (%)

ASA-PS, American Society of Anesthesiology physical status

^a^Arrhythmia requiring medical therapy such as atrial fibrillation and atrioventricular block

^b^Weekly consumption of alcohol more than 150 mL or in equivalent dosage

^c^A self-rated questionnaire that assesses sleep quality within 1 month which ranges from 0 to 21 with higher score for worse sleep quality

^d^Score ranges from 20 to 80 with higher score indicating heavier anxiety

^e^Includes a 4-item chronic pain severity scale and a 7-item pain-related-function interference scale. The mean scores range from 0 to 10 with higher score indicating heavier intensity or worse pain-related function

^f^Assessment of daily living activities. Score ranges from 0 to 100 with higher score for better activity

**Table 2:** Perioperative data

	Remimazolam group (*n* = 54)	Routine group (*n* = 54)	P
Study center, *n*			> 0.999
Site 1	39	39	
Site 2	15	15	
Type of surgery, *n*			**0.039**
Total knee arthroplasty	49 (90.7%)	41 (75.9%)	
Total hip arthroplasty	5 (9.3%)	13 (24.1%)	
Intraoperative drugs			
Dexmedetomidine, μg^a^	–	35.0 (25.5, 50.0) (*n* = 45)	–
Bupivacaine, mg^b^	12.6 ± 2.3 (*n* = 52)	12.3 ± 2.2 (*n* = 53)	0.560
Epidural lidocaine, *n*	15 (28.8%) (*n* = 52)	19 (35.8%) (*n* = 53)	0.443
Epidural ropivacaine, *n*	5 (9.6%) (*n* = 52)	10 (18.9%) (*n* = 53)	0.176
Use of tropisetron, *n*	46 (85.2%)	49 (90.7%)	0.375
Use of vasopressors, *n*^c^	8 (14.8%)	7 (13.0%)	0.781
Duration of anesthesia, min	180 ± 35	173 ± 41	0.326
Duration of surgery, min)	118 ± 36	116 ± 36	0.825
TWA BIS from incision to the end of surgery^d^	77 ± 7	85 ± 9	** < 0.001**
Cumulative time of BIS, min^e^			
≥ 81	11 (0, 26)	98 (69, 113)	** < 0.001**
70–80	72 (59, 97)	9 (1, 18)	** < 0.001**
≤ 69	11 (2, 28)	1 (0, 8)	** < 0.001**
Intraoperative fluid balance			
Total intraoperative infusion, ml	1500 (1275, 1800)	1500 (1300, 1800)	0.477
Allogenic blood transfusion, *n*^f^	7/54 (13%)	12/54 (22%)	0.206
Estimated blood loss, ml	100 (50, 150)	100 (50, 200)	0.311
Intraoperative urine output, ml	400 (200, 763)	400 (250, 525)	0.810
Ultrasound-guided nerve block, *n*			0.064
Femoral nerve block	49 (90.7%)	42 (77.8%)	
Fascia iliaca compartment block	5 (9.3%)	12 (22.2%)	
Types of NSAIDS, *n*^g^			0.691
Flurbiprofen axetil	41 (76%)	40 (74%)	
Parecoxib	2 (3.7%)	4 (7.4%)	
Ketorolac tromethamine	11 (20.4%)	10 (18.5%)	
Postoperative use of oral opioids, *n*^h^			
Surgery day	21 (38.9%)	28 (51.9%)	0.176
First day	26 (48.1%)	29 (53.7%)	0.564
Second day	31 (57.4%)	29 (53.7%)	0.699
Postoperative use of sedatives, *n*^i^			
Night at surgery	2 (3.7%)	6 (11.1%)	0.270
First night	10 (18.5%)	6 (11.1%)	0.279
Second night	5 (9.3%)	5 (9.3%)	> 0.99
Dosage of sufentanil by PCIA, μg			
Surgery day	26 (20, 35)	26 (20, 38)	0.692
First day	61 (48, 78)	59 (49, 74)	0.614
Second day	100 (74, 111)	97 (81, 100)	0.454

*P* values in bold indicate < 0.05

Data are presented as mean ± SD, median (interquartile range), or number (%)

TWA, time-weighted average; BIS, bispectral index; PCIA, patient-controlled intravenous analgesia

^a^Used in routine care group

^b^Used for spinal anesthesia

^c^Including ephedrine, metaraminol, and phenylephrine

^d^BIS value was recorded at 1 min interval from initial administration of trial drug to end of surgery. TWA BIS was calculated as the summation of BIS values multiplied by the referred duration, and divided by the whole recording duration

^e^Calculated as the accumulated time of BIS values from incision to the end of surgery within predefined references

^f^Including packaged red blood cell and fresh frozen plasm

^g^NSAIDs was given at end of surgery and then at 12 h interval until postoperative 72 h including flurbiprofen, parecoxib, and ketorolac

^h^Including oral oxycodone acetaminophen, pentazocine, and tramadol

^i^Including estazolam, oxazepam, and zolpidem

### Primary outcome

The RCSQ score at surgery night was 59 (28, 75) in remimazolam group which was comparable with 53 (28, 67) in routine care group (median difference 6, 95% CI − 6 to 16, *P* = 0.315, Table 3 and Supplemental Fig. S1). This result was also verified in per protocol analysis [62 (27, 77) vs. 52 (28, 66), MD = 6 (− 4 to 16), *P* = 0.211]. In post-hoc analysis, patients in remimazolam group had similar RSCQ in comparison with those treated with dexmedetomidine [62 (27, 77) vs. 54 (28, 70), MD = 4 (− 8 to 16), *P* = 0.494, Table 3 and Supplemental Table S1].

**Table 3:** Efficacy outcomes

	Remimazolam group group (*n* = 54)	Routine group (*n* = 54)	Estimated effect size (95% CI)	*P*^a^
*Primary outcome*				
RCSQ at surgery night, score^b^				
Intention-to-treat	59 (28, 75)	53 (28, 67)	MD = 6 (− 6, 16)	0.315
Per protocol	62 (27, 77) (*n* = 52)	52 (28, 66) (*n* = 53)	MD = 6 (− 4, 16)	0.211
*Secondary outcomes*				
RCSQ after surgery, score^b^				
First night	69 (56, 85)	70 (54, 80)	MD = 2 (− 4, 10)	0.472
Second night	80 (68, 87)	76 (64, 84)	MD = 4 (0, 10)	0.066
NRS pain intensity at rest, score^c^				
First day	1 (0, 3)	0 (0, 2)	MD = 0 (0, 1)	0.114
Second day	1 (0, 3)	1 (0, 2)	MD = 0 (0, 1)	0.206
Third day	1 (0, 3)	0 (0, 2)	MD = 0 (0, 0)	0.313
Postoperative nausea and vomiting, *n*	10 (18.5%)	7 (13.0%)	RR = 1.43 (0.59, 3.48)	0.428
Major complications, *n*^d^	1 (1.9%)	3 (5.6%)	RR = 0.33 (0.04, 3.11)	0.618
Length of in-hospital stay after surgery, day	7 (6, 10)	7 (7, 11)	HR = 1.16 (0.79, 1.70)	0.449

Data are presented as median (interquartile range) or number (%)

MD, median difference or mean difference; HR, hazard ratio; RR, relative risk; RCSQ, Richards Campbell Sleep Questionnaire; NRS, Numeric Rating Scale

^a^Calculated as BIS-guided group versus or minus routine group

^b^The RCSQ involves five domains including sleep depth, sleep latency, awakenings, returning to sleep, and sleep quality. Overall RCSQ sleep score is defined as the mean value of above five domains and it ranges from 0 to 100 with higher score for better sleep

^c^Pain intensity at rest was assessed by numeric rating scale (11-point scale, 0 for no pain and 10 for the worst pain)

^d^Complications requiring medical interventions (i.e., Clavien–Dindo classification 2 and above) within postoperative 30 days. One patient in BIS-guided group suffered lower limbs venous thrombosis. Three patients in routine group suffered complications including cardiac injury, new-onset atrial fibrillation, and urinary tract infection

Unbalanced variables between two groups (history of hypertension and total hip replacement surgery) and factors with clinical significance (age, female, preoperative PSQI, SAS, BPI and intraoperative TWA BIS) were considered as confounders. After adjustment, multivariable generalized linear regression showed that remimazolam was not associated with RCSQ score at surgery night (β 1.80, 95% CI − 9.46 to 13.06, *P* = 0.754, Supplemental Table S2). Higher preoperative PSQI score (indicating poorer sleep quality) was associated with decrement of RCSQ score (β − 1.67, 95% CI − 3.19 to − 0.14, *P* = 0.032).

### Secondary outcomes

RCSQ score at postoperative first night [69 (56, 85) vs. 70 (54, 80), *P* = 0.472] and second night [80 (68, 87) vs. 76 (64, 84), *P* = 0.066] were comparable between two groups (Table 3). At second night, patients in remimazolam group had slightly higher scores in domains of return to sleep and sleep quality (*P* = 0.040 and 0.046, respectively, Supplemental Table S3). Pain intensity was comparable between two groups during postoperative 3 days (Table 3). The incidences of PONV and major complications did not differ statistically between study groups. Patients in two groups had similar duration of postoperative in-hospital stay (Table 3).

### Exploratory outcomes

**Fig. 2:**
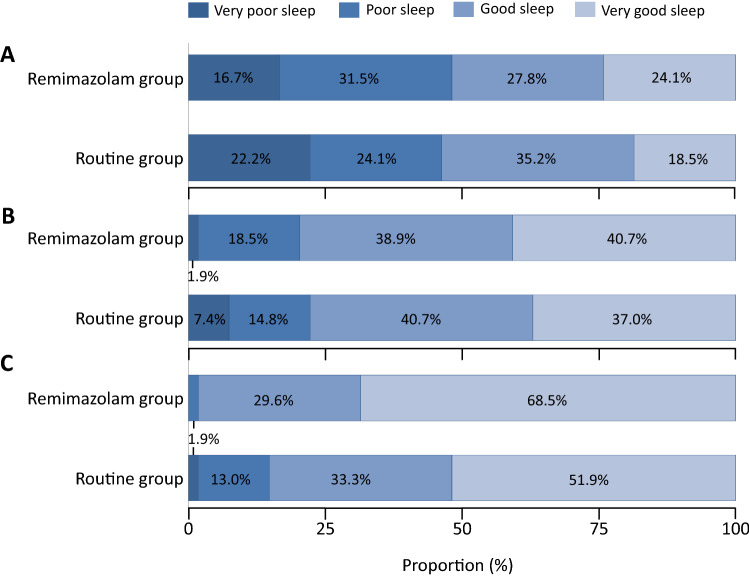
Richards Campbell Sleep Questionnaire scores distribution between two groups of the first 3 nights after surgery. Horizontal stacked bar graphs show Richards Campbell Sleep Questionnaire score distribution between BIS-guided sedation group and routine care group of **A** night of the surgery, **B** first night after surgery and **C** second night after surgery. Bars are labelled with proportions

Classifications of RCSQ quality at each night were presented in Fig. 2. Constituent ratio of sleep quality was comparable at surgery night and postoperative first night. At postoperative second night, patients in remimazolam group had higher proportion of good sleep than routine group (98.1% vs. 85.2%, *P* = 0.015, Fig. 2). Subgroup analysis showed no significant differences of RCSQ scores between high dose and low dose of drug both in remimazolam group and dexmedetomidine group (Table S4).

### Safety outcomes

Patients in routine group had slightly higher incidence of intraoperative hypotension but without statistical significance (16.7% vs. 14.8%, *P* > 0.99). There was also no statistical significance in other adverse events including hypertension, bradycardia, tachycardia, respiratory depression, nausea and vomiting (Table 4).

**Table 4:** Adverse events

	Remimazolam group (*n* = 54)	Routine group (*n* = 54)	*P*
Intraoperative period			
Hypotension, *n*^a^	8 (14.8%)	9 (16.7%)	> 0.99
Hypertension, *n*^b^	2 (3.7%)	4 (7.4%)	0.678
Bradycardia, *n*^c^	1 (1.9%)	2 (3.7%)	> 0.99
Tachycardia, *n*^d^	2 (3.7%)	0 (0%)	0.495
Desaturation, *n*^e^	1 (1.9%)	1 (1.9%)	> 0.99
Nausea, *n*	0 (0%)	2 (3.7%)	0.495
Vomiting, *n*	0 (0%)	0 (0%)	–
Postoperative period			
Desaturation, *n*^e^	0 (0%)	0 (0%)	–
Nausea, *n*	2 (3.7%)	3 (5.6%)	> 0.99
Vomiting, *n*	0 (0%)	0 (0%)	–

Data are presented as number (%)

^a^Defined as systolic blood pressure < 90 mmHg or a decrease of > 20% from baseline

^b^Defined as systolic blood pressure > 160 mmHg or an increase of > 20% from baseline

^c^Defined as heart rate < 50 beat per minute or a decrease of > 20% from baseline

^d^Defined as heart rate > 100 bpm or an increase of > 20% from baseline

^e^Defined as pulse oxygen saturation < 90% or an absolute decrease of > 5% from baseline

## Discussion

The present study demonstrated that intraoperative remimazolam sedation (kept at BIS 70–80), compared with those mainly treated with dexmedetomidine, did not significantly improve postoperative sleep quality in elderly patients receiving total joint arthroplasty under neuraxial anaesthesia. However, remimazolam is proved to be effective and safe for moderate sedation in elderly during reginal anaesthesia.

Nowadays, sleep disturbance after surgery has become a major concern especially in elderly patients. In this study, very poor or poor sleep quality happened in about half of patients at surgery night and its severity gradually alleviated during the following two nights, which was in line other studies [3, 18]. We employed PSQI for baseline sleep quality assessment and RCSQ for postoperative nightly assessment in the study. Both of the two instruments had been validated in Chinese patients [11, 14, 15]. PSQI is considered as a reliable scale to assess chronic sleep quality within 1 month [11]. For acute sleep quality assessment, numeric rating sleep scale was commonly used, but this method is merely a rough report of subjective sleep quality. Compared with PSQI and NRS scale, RCSQ reflects the acute change of sleep quality and includes five dimensional assessments of patient’s sleep [14, 15]. Recent studies have validated the effectiveness and reliability of RCSQ in general surgery patients [16].

In the present study, remimazolam was titrated to maintain sedation depth at BIS 70–80. This is based on the result of previous study in which intraoperative midazolam with BIS of 75–80 improved postoperative sleep quality [8]. Benzodiazepines is the mainstream for sedation in orthopaedic patients and account for 80% of sedatives in patients undergoing total joint arthroplasty [19], although with concerns on increased risks of delirium, apnoea and hypoxia [9, 20]. These adverse effects may be partially attributed to its relative long-acting time from several hours to days [19]. Remimazolam is a new agent of benzodiazepine family with ultra-short half-life time [21]. Thus, it preserves significant advantage in better recovery of consciousness and psychomotor performance including task execution and memory after procedure sedation [22].

The effect of remimazolam on sleep quality might be underestimated by the relatively small sample size of our study. Beyond anxiety, sleep disturbance arises from multiple etiologies including pain intensity, environmental noise, and procedures of medical therapy [2, 3, 23]. On the other hand, the ultra-short half-time of remimazolam may also limit its therapeutic effect. Thus, for sample size calculation, we conservatively proposed an assumption of a 20% increment in RCSQ score by remimazolam. Although no statistical significance was observed between two groups, median RCSQ score at surgery night was about 59 in remimazolam group and 53 in routine group which was close to our assumption (i.e., 60 vs. 50). This result indicated that intraoperative remimazolam may be used to improve sleep quality. However, further studies with large sample size are needed to validate this assumption, especially if postoperative remimazolam during surgery night will have better performance. For example, a randomized controlled study reported that patients who received zolpidem for 2 weeks after total knee arthroplasty had better sleep quality and lower pain scores [24].

Sedation strategy in routine group is based on daily clinical practice in two participating centres because of the following concerns. First, sedation at the discrete of patient’s demand and anaesthesiologist’s advice is the most common approach in most centres. The data originated from routine group are close to real-world practice and this facilitates the generalization of our result. Second, most sedatives were given in terms of clinical experience but not BIS-guided sedation in daily practice. Thus, BIS readings in routine group were masked to attending anaesthesiologists, which helps to alleviate the influence of BIS monitoring on daily practice. Third, previous evidence reported that intraoperative dexmedetomidine at 0.2–0.4 μg/kg/h might decrease the incidence of severe sleep disturbance at surgery night [25], and low dose dexmedetomidine was commonly used for sedation in our routine practice. In the present study, the median dosage of dexmedetomidine in routine group was 0.3 μg/kg/h, possibly optimizing postoperative sleep quality to some extent. However, we noticed that about half of these patients still complained sleep disturbance. This result is partially inconsistent with previous studies which reviewed the protective effect of dexmedetomidine on sleep [7]. As we discussed above, sleep disturbance is multifactorial. Further studies are needed to verify multidiscipline interventions on sleep quality, not only by sedatives.

We noticed that patients in routine group have lighter sedation depth in comparison with remimazolam group. We took two steps to evaluate the effect of BIS value on outcome. Multivariable linear regression was firstly administrated to adjust TWA BIS and other confounders. Second, a post-hoc analysis was employed to compare outcomes in patients with remimazolam and dexmedetomidine after exclusion of 9 patients without dexmedetomidine in routine group. The result was consistent with intention-to-treat analysis. Based on clinical experience, anaesthesiologists are prone to maintain lighter sedation in avoidance of adverse effects such hypotension and bradycardia by dexmedetomidine. In this study, we also observed slightly higher incidence of hypotension in routine care group but without statistical significance. However, the effect of sedation depth on patient’s prognosis is uncertain till now. In patients undergoing hip surgery, BIS-guided propofol light sedation (BIS > 80 versus deep BIS < 50) was associated with 50% reduction in postoperative delirium and 1 year mortality [26, 27]. But these results were not supported by the STRIDE trials which employed modified Observer's Assessment of Alertness/Sedation score (MOAA/S) as target of depth (lighter sedation 3–5 vs. heavier sedation 0–2) [28, 29].

To date, few studies have evaluated the effectiveness and safety of remimazolam infusion for sedation in elderly patients during spinal anaesthesia. Our study reported that a loading dose followed by continuous infusion of remimazolam could be safely and effectively used for intraoperative targeted sedation without increasing risks of adverse outcomes such as hypotension and respiratory depression.

Preoperative sleep disturbance is associated with postoperative sleep and pain intensity [30, 31]. Our study also found that poor preoperative sleep quality (higher PSQI scores) was associated with postoperative sleep disturbance. This indicates that improvement of preoperative sleep quality may alleviate the risk of postoperative sleep disturbance.

Our study had several limitations. First, architecture of sleep was monitored by RCSQ but not polysomnography. It is true that polysomnography represents a gold-standard tool for evaluation of sleep disorders [32], yet it was impractical in perioperative settings and may interfere with patient’s medical care, even sleep quality. Previous study showed that RCSQ had high consistence with polysomnography [33]. Second, the sample size was underpowered. Sample size calculation was based on a SD of 15 in pilot observation. But the actual SD was about 26. Further studies with larger sample size should be considered. Third, sedation depth was monitored by BIS. Previous studies showed that BIS may be inappropriate for monitoring dexmedetomidine sedation [34]. Objective scales such as MOAA/S may be better. However, frequent assessments will interrupt sedation continuity. This is not friendly to patient’s experiences. Another question is the effect of sedation depth on sleep quality. We employed multivariable linear regression and post hoc analysis to explore the association. Fourth, our study only focused on elderly patients undergoing orthopaedic surgery. Further studies are needed to identify the generalizability in other populations.

In conclusion, for elderly patients undergoing total joint arthroplasty under neuraxial anaesthesia, intraoperative remimazolam can be safely and effectively used for sedation, but its effect on postoperative sleep quality is still uncertain.

## Supplementary Information

Below is the link to the electronic supplementary material.

**Fig. S1**: Richards Campbell Sleep Questionnaire scores of the first 3 nights after surgery. The box and whiskers plots show medians, interquartile ranges and outer ranges, and individual points mean mild outliers (o, which are outside 1.5 times of interquartile range). (PDF 201 KB)

**Table S1**: Primary and secondary Outcomes among patients receiving remimazolam and dexmedetomidine. **Table S2**: Factors associated with score of Richards Campbell Sleep Questionnaire at surgery night. **Table S3**: The scores in each domain of Richards Campbell Sleep Questionnaire. **Table S4**: The differences of Richards Campbell Sleep Questionnaire scores between high dose or low dose of dexmedetomidine and remimazolam. (DOCX 26 KB)

## Data Availability

The dataset analysed in the current study is available from the corresponding author on reasonable request.
