# Supplementary figures and images for: Effect of intraoperative remimazolam on postoperative sleep quality in elderly patients after total joint arthroplasty: a randomized control trial
Source: J Anesth. 2023 Apr 13;37(4):511–21. doi: 10.1007/s00540-023-03193-5 (PMC10390348; doi:10.1007/s00540-023-03193-5)

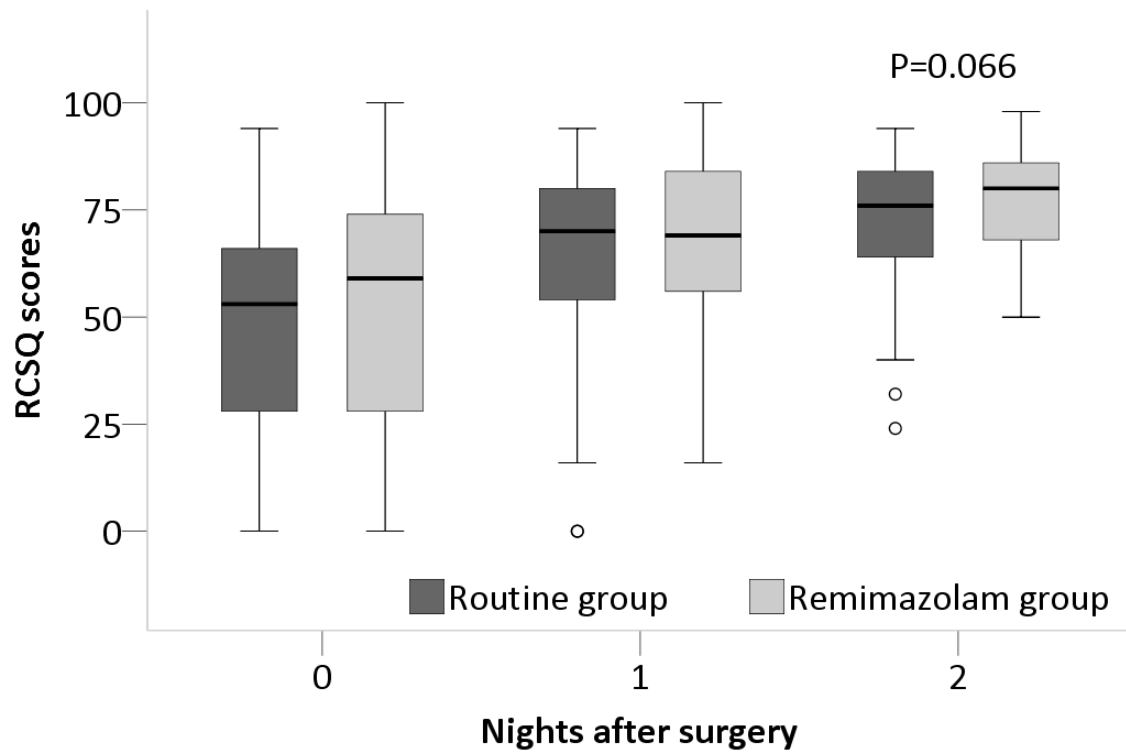

Supplement: Supplementary file 1 — Fig. S1: Richards Campbell Sleep Questionnaire scores of the first 3 nights after surgery. The box and whiskers plots show medians, interquartile ranges and outer ranges, and individual points mean mild outliers (o, which are outside 1.5 times of interquartile range). (PDF 201 KB) [file 540_2023_3193_MOESM1_ESM.pdf]
