# Supplementary material for: Effect of intraoperative remimazolam on postoperative sleep quality in elderly patients after total joint arthroplasty: a randomized control trial
Source: J Anesth. 2023 Apr 13;37(4):511–21. doi: 10.1007/s00540-023-03193-5 (PMC10390348; doi:10.1007/s00540-023-03193-5)
Supplement: Supplementary file 2 — Table S1: Primary and secondary Outcomes among patients receiving remimazolam and dexmedetomidine. Table S2: Factors associated with score of Richards Campbell Sleep Questionnaire at surgery night. Table S3: The scores in each domain of Richards Campbell Sleep Questionnaire. Table S4: The differences of Richards Campbell Sleep Questionnaire scores between high dose or low dose of dexmedetomidine and remimazolam. (DOCX 26 KB) [file 540_2023_3193_MOESM2_ESM.docx]

**Table S1. Primary and secondary Outcomes among patients receiving remimazolam and dexmedetomidine**

|  | **BIS-guided sedation with remimazolam**  (n=52) | **Routine care with dexmedetomidine**  (n=45) | **P** |
| --- | --- | --- | --- |
| **Primary outcome** |  |  |  |
| RCSQ at surgery night, score **^a^** | 62 (27, 77) | 54 (28, 70) | 0.494 |
| **Secondary outcomes** |  |  |  |
| RCSQ after surgery, score **^a^** |  |  |  |
| First night | 69 (57, 84) | 70 (54, 81) | 0.794 |
| Second night | 80 (69, 88) | 76 (65, 86) | 0.229 |
| NRS pain intensity at rest, score **^b^** |  |  |  |
| First day | 1 (0, 3) | 0 (0, 2) | 0.080 |
| Second day | 2 (0, 3) | 1 (0, 2) | 0.167 |
| Third day | 1 (0, 3) | 0 (0, 2) | 0.435 |
| Postoperative nausea and vomiting, n | 10 (19.2%) | 7 (15.6%) | 0.635 |
| Major complications, n **^c^** | 1 (1.9%) | 1 (2.2%) | >0.999 |
| Length of in-hospital stay after surgery, day | 8 (6, 10) | 7 (6,10) | 0.973 |

Data are presented as median (interquartile range) or number (%).RCSQ=Richards Campbell Sleep Questionnaire; NRS=Numeric Rating Scale.

**^a^** The RCSQ involves five domains including sleep depth, sleep latency, awakenings, returning to sleep, and sleep quality. Overall RCSQ sleep score is defined as the mean value of above five domains and it ranges from 0 to 100 with higher score for better sleep.

**^b^** Pain intensity at rest was assessed by numeric rating scale (11-point scale, 0 for no pain and 10 for the worst pain).

**^c^** Complications requiring medical interventions (i.e., Clavien-Dindo classification 2 and above) within postoperative 30 days. One patient in BIS-guided group and one in routine group suffered lower limbs venous thrombosis.

**Supplementary table S2. Factors associated with score of Richards Campbell Sleep Questionnaire at surgery night**

| **Variables** | **β** | **95% confidence interval** | | **P value ^a^** |
| --- | --- | --- | --- | --- |
|  |  | **Lower limit** | **Upper limit** |  |
| Remimazolam sedation (yes) **^b^** | 1.80 | -9.46 | 13.06 | 0.754 |
| Age (per year increase) | -0.39 | -1.38 | 0.60 | 0.441 |
| Female (yes) | -5.30 | -16.37 | 5.78 | 0.349 |
| Hypertension (yes) | 2.88 | -7.69 | 13.45 | 0.593 |
| SAS (per score increase) **^c^** | 0.29 | -0.87 | 1.45 | 0.629 |
| PSQI (per score increase) **^d^** | **-1.67** | **-3.19** | **-0.14** | **0.032** |
| BPI (per score increase) ^e^ | -0.66 | -4.81 | 3.49 | 0.755 |
| Total hip replacement (yes) | 0.21 | -13.66 | 14.07 | 0.977 |
| TWA BIS value | -0.22 | -0.83 | 0.39 | 0.474 |

SAS= Self-rating anxiety scale; PSQI= Pittsburgh Sleep Quality Index; BPI= Brief Pain Inventory. TWA BIS= time weighed average Bispectral index; P values in bold indicate <0.05.

**^a^** Analyzed by multivariable generalized linear regression analyses. Unbalanced variables between two groups (history of hypertension and type of surgery) and clinically significant factors (age, female, preoperative PSQI, SAS, BPI and TWA BIS value) were considered as confounders.

**^b^** Light sedation (BIS 70-80) was targeted for intraoperative sedation by infusion of remimazolam.

**^c^** Higher score indicated heavier anxiety.

**^d^** Higher score indicated poorer sleep quality within recent one month.

**^e^** Higher score indicated heavier intensity and worse pain-related function.

**Supplementary table S3. The scores in each domain of Richards Campbell Sleep Questionnaire**

|  | **BIS-guided group (n=54)** | **Routine group (n=54)** | **P** |
| --- | --- | --- | --- |
| **At night of surgery** |  |  |  |
| Sleep depth **^a^** | 60 (30, 80) | 50 (30, 80) | 0.416 |
| Falling asleep **^b^** | 50 (30, 80) | 50 (28, 80) | 0.831 |
| Awakening **^c^** | 50 (30, 70) | 45 (30,70) | 0.233 |
| Return to sleep **^d^** | 60 (30, 80) | 50 (20,70) | 0.268 |
| Sleep quality **^e^** | 60 (30, 80) | 50 (30,70) | 0.309 |
| Noise **^f^** | 80 (60, 100) | 90 (70,100) | 0.299 |
| **At first night after surgery** |  |  |  |
| Sleep depth **^a^** | 70 (50, 82) | 70 (50, 80) | 0.941 |
| Falling asleep **^b^** | 80 (58, 90) | 70 (58, 83) | 0.438 |
| Awakening **^c^** | 70 (50, 80) | 70 (48, 80) | 0.445 |
| Return to sleep **^d^** | 70 (50, 90) | 70 (50,80) | 0.251 |
| Sleep quality **^e^** | 70 (60, 90) | 70 (50,80) | 0.155 |
| Noise **^f^** | 90 (70, 100) | 90 (80, 100) | 0.318 |
| **At second night after surgery** |  |  |  |
| Sleep depth **^a^** | 80 (70, 90) | 80 (68, 90) | 0.987 |
| Falling asleep **^b^** | 80 (70, 90) | 80 (70, 90) | 0.129 |
| Awakening **^c^** | 80 (60, 90) | 70 (50, 80) | 0.097 |
| Return to sleep **^d^** | 80 (70, 90) | 75 (50, 90) | 0.040 |
| Sleep quality **^e^** | 80 (70, 90) | 80 (60, 83) | 0.046 |
| Noise **^f^** | 90 (80, 100) | 90 (80, 100) | 0.499 |

Data are presented as median (interquartile range).

**^a^** Each of the following domain was assessed by using a 100-mm visual analog scale in which a higher score is better. Scores of a question: “My sleep last night was: Deep sleep …Light sleep”

**^b^** Scores of a question: “Last night, the first time I got to sleep, I: Fell asleep almost immediately… Just never could fall asleep”.

**^c^** Scores of a question: “Last night, I was: Awake very little… Awake all night”.

**^d^** Scores of a question: “Last night, when I woke up or was awakened, I: Got back to sleep immediately… Couldn’t get back to sleep”.

**^e^** Scores of a question: “I would describe my sleep las night as: A good night’s sleep… A bad night’s sleep”.

**^f^** Scores of a question: “I would describe the noise level last night as: Very quiet… Very noisy”.

**Supplementary table S4. The differences of Richards Campbell Sleep Questionnaire scores between high dose or low dose of dexmedetomidine and remimazolam.**

| **Remimazolam ^a^** | **Low dose (≤ 25mg)**  **(n=27)** | **High dose (>25mg)**  **(n=25)** | **Median difference**  **(95% CI) ^b^** | **P** |
| --- | --- | --- | --- | --- |
| RCSQ at surgery night, score | 62 (30, 78) | 50 (26, 77) | -2 (-16, 14) | 0.797 |
| RCSQ at first night after surgery, score ^b^ | 72 (62, 88) | 68 (51, 80) | -4 (-14, 6) | 0.469 |
| RCSQ at second night after surgery, score ^b^ | 82 (70, 90) | 80 (68, 86) | -2 (-8, 4) | 0.575 |
| **Dexmedetomidine ^a^** | **Low dose (≤ 35μg)**  **(n=23)** | **High dose (>35μg)**  **(n=22)** | **Median difference**  **(95% CI) ^b^** | **P** |
| RCSQ at surgery night, score | 54 (28, 60) | 58 (28, 80) | 10 (-10, 24) | 0.301 |
| RCSQ at first night after surgery, score ^b^ | 76 (54, 80) | 70 (53, 87) | -2 (-14, 12) | 0.865 |
| RCSQ at second night after surgery, score ^b^ | 84 (66, 88) | 75 (64, 81) | -4 (-12, 4) | 0.237 |

**^a^** Patients were divided into high dose and low dose groups by median values of remimazolam and dexmedetomidine (i.e. ≤ median dose or >median dose).

**^b^** Calculated as high dose group minus low dose group.
